# Claspin as a biomarker of human papillomavirus-related high grade lesions of uterine cervix

**DOI:** 10.1186/1479-5876-10-132

**Published:** 2012-06-25

**Authors:** Maria Benevolo, Antonio Musio, Amina Vocaturo, Maria Gabriella Donà, Francesca Rollo, Irene Terrenato, Mariantonia Carosi, Edoardo Pescarmona, Giuseppe Vocaturo, Marcella Mottolese

**Affiliations:** 1 Pathology Department, Regina Elena Cancer Institute, Via Elio Chianesi 53, 00144, Rome, Italy; 2 Istituto di Ricerca Genetica e Biomedica, Consiglio Nazionale delle Ricerche, Pisa, Italy; 3 Istituto Toscano Tumori, Florence, Italy; 4 Sexually Transmitted Infections (STI) Unit, San Gallicano Dermatological Institute, Rome, Italy; 5 Epidemiology Department, Regina Elena Cancer Institute, Rome, Italy; 6 Oncologic Gynaecology Department, Regina Elena Cancer Institute, Rome, Italy

**Keywords:** Claspin, Human papillomavirus, Uterine cervix, Biomarker

## Abstract

**Background:**

Claspin is a nuclear protein involved in DNA replication and damage response and is a key mediator for the S-phase checkpoint. Claspin expression is significantly high in several human solid tumors. Furthermore, high levels of claspin have been found in cervical cancer cell lines. Nevertheless, no data are available regarding claspin expression in cervical tissues.

**Methods:**

In order to investigate whether claspin immunoreactivity is related to the lesion severity and High-Risk (HR) HPV infection, we analyzed claspin expression by immunohistochemistry in a series of cervical biopsies which represent the steps occurring during cervical carcinogenesis (normal tissues, Cervical Intraepithelial Neoplasias 1, 2 and 3, Squamous Cell Carcinomas). All patients also had a cervico-vaginal sample for HPV testing, collected immediately before the colposcopy-guided biopsy. The HR-HPV DNA detection was performed by the HR-HPV Hybrid Capture 2 test. HPV genotyping was performed using the Linear Array HPV Genotyping Test.

**Results:**

Our results evidenced a constant and significant increase of the rate of claspin positivity from the normal tissues to carcinomas (pχ^2^_trend_ < 0.0001). In fact, the normal tissues displayed either no or faint claspin immunoreactivity, whereas a moderate/high positivity was observed in 16% of the CIN1, 76% of the CIN2, 87.5% of the CIN3 and 93.3% of the cancers. Moreover, we found a statistically significant correlation between claspin expression and HR-HPV infection (pχ^2^ < 0.0001), irrespective of the genotype. Finally, we demonstrated the feasibility of claspin immunostaining in cervical cytology.

**Conclusions:**

Our findings indicate that *in vivo* claspin expression is significantly related to HR-HPV infection and lesion grade both in histological and cytological samples. Therefore, the analysis of claspin expression could be clinically relevant in the diagnosis of HPV-related cervical lesions, in particular when applied to cervico-vaginal cytology. Moreover, giving information on the proliferation rate of each lesion, claspin immunostaining may contribute to the evaluation of progression risk, thus being helpful in patient management. Nevertheless, only large prospective studies may clarify the true clinical usefulness of claspin expression in distinguishing lesions with different progression potential.

## Background

Claspin is a nuclear protein involved in both DNA replication and damage response and is a key mediator for the S-phase checkpoint. Its main function is to facilitate phosphorylation of the effector kinase Chk1 by the sensor kinase ATR in response to replication stresses [1-3]. Inhibition of claspin, as well as ATR and Chk1, greatly reduces cell survival, likely inducing alterations in cell cycle checkpoints and DNA repair systems [4]. These alterations may lead to genomic instability triggering cancer development [5,6]. However, the expression of claspin significantly increases in several human solid tumors such as in colon, lung, bladder and breast cancer [7]. Despite these findings, to date no data are available concerning claspin expression in human cervical carcinogenesis which represents a paradigmatic model of cancer development. Cervical cancer, in fact, arises via a series of well-defined intermediate lesions throughout various steps of cellular atypia and its cause, i.e. the High Risk Human Papillomavirus (HR-HPV) infection, is well established. The oncogenic potential of HR-HPV lies in the two major viral oncoproteins, E6 and E7, which bind to and modulate a number of different cellular proteins, interfering with almost all the cellular processes and inducing genomic instability and cell proliferation. Interestingly, recent *in vitro* data showed high claspin baseline level in cervical cancer cell lines. In addition, the cell lines expressing the HPV-E7 oncoprotein also showed an increased claspin turnover [8].

Therefore, in addition to viral markers, such as E6/E7 mRNA or protein detection [9,10], cellular molecules related to cell cycle control, may be worth of investigation in cervical pathology. Several host proteins, such as p16 and Ki67 [11-13], have been studied, possibly representing novel biomarkers which are strictly linked to the host changes induced by HPV. Yet, none of them has been introduced in clinical guide lines so far, although p16 expression is currently used by many pathologists.

In the present study, we addressed the question of whether the expression of claspin is associated to human uterine cervix transformation *in vivo* and, as a consequence, if it could be used as a putative biomarker of dysplasia and/or HR-HPV infection. To this end, we firstly analyzed claspin expression by western blotting in a series of both HPV negative and positive non tumorigenic and tumorigenic cell lines. Furthermore, we determined immunohistochemical claspin distribution, in parallel with HR-HPV DNA status, in a retrospective series of human cervical biopsies which encompass a representative spectrum of dysplastic and neoplastic cervical lesions, aiming at investigating whether claspin expression correlated with the grade of the lesions and HR-HPV infection *in vivo*.

Finally, in the knowledge that identifying molecular alterations in cytological specimens might improve the detection of cervical lesions at higher risk of transformation in both primary screening and triage settings, we evaluated claspin distribution in a subset of available cytological samples, including negative, low and high grade squamous intraepithelial lesions, collected in parallel with the cervical biopsies.

## Materials and methods

### Cell lines

The human cell lines NHEK, HK-168, SiHa, CaSki, and HeLa were used. They were kindly provided by Dr. Federico De Marco (Regina Elena Cancer Institute, Rome, Italy). NHEK is a HPV negative human keratinocyte diploid cell line; HK-168 is a non tumorigenic cell line derived from primary human keratinocytes transfected with HPV16 and containing about 1–2 viral copies/aploid genome [14]. SiHa, CaSki and HeLa are cervical carcinoma-derived cell lines (the SiHa and CaSki from squamous cancer and the HeLa from an adenocarcinoma) containing HPV sequences (SiHa: 1 HPV16 genome copy per cell; CaSki: 500–600 HPV16 genome copies per cell; HeLa: 10–50 HPV18 genome copies per cell).

NHEK and HK-168 were cultured in a K-SMC (Gibco, Invitrogen, Milan, Italy) definite medium while SiHa, CaSki and HeLa were cultured in DMEM supplemented with 10% of fetal bovine serum and antibiotics (penicillin 100 U/mL, and streptomycin 100 μg/mL).

### Western blotting

Analysis with rabbit polyclonal antibody against claspin (Bethyl Laboratories, Montgomery, TX, USA) was performed according to a published protocol [5]. Briefly, the samples were boiled in sample buffer and separated by SDS-PAGE. The proteins were, then, transferred to a nitrocellulose membrane (Amersham Pharmacia Biotech, Freiburg, Germany) and incubated with the primary antibody. After removing the unbound primary antibody, the membranes were incubated with a secondary antibody-peroxidase conjugate (Sigma Chemical Co., MO, USA), processed for detection by chemiluminescence (Amersham), and imaged on Biomax film (Kodak, Milan, Italy). A rabbit polyclonal antibody against Actin (Santa Cruz Biotechnology, CA, USA) was used as a loading control.

### Histological samples

This study was reviewed by the Ethics Committee of the Regina Elena Cancer Institute. We selected 80 cases from the Regina Elena Cancer Institute files, which had been collected between June 2006 and July 2009, submitted to a colposcopy-guided cervical biopsy immediately after a cervico-vaginal sampling for HPV testing. All histological slides were independently reviewed by two experienced pathologists and an adjudicated final diagnosis was established. Of the 80 formalin fixed biopsies, 9 had no relevant lesions (herein referred to as WNL: within normal limits), 19 were diagnosed as Cervical Intraepithelial Neoplasia (CIN) 1, 21 as CIN2, 16 as CIN3 and 15 as invasive Squamous Cell Carcinomas (SCC). For the purpose of our study, the WNL and CIN1 diagnoses were referred to as CIN2-, while CIN2, CIN3 and SCC were referred to as CIN2+. Therefore, on the basis of this classification, we analyzed 28 CIN2- and 52 CIN2+ cases. This sample size was adequate to detect a significant difference at 1% level with a statistical power of 90%, according to an *a priori* power calculation based on data from our laboratory regarding Ki67 reactivity in cervical pathology (data not shown). In fact, given that Ki67 is a well known proliferation marker which is widely applied in solid tumors [15], we expected a similar distribution of immunoreactivity for claspin. The sample size calculation identified a minimum number of 54 patients, 27 per CIN2- group and 27 per CIN2+ group.

### Cytological samples

Of the 80 cervico-vaginal specimens collected in PreservCyt (Hologic, Rome, Italy) for HPV testing, 19 had a residual sample which was sufficient to obtain thin layer slides which were adequate for both morphological and immunocytochemical analyses. The thin layer slides were prepared using the ThinPrep 2000 System (Hologic), following the manufacturer’s instructions. Papanicolau slides were interpreted independently from all other findings, and classified according to the Bethesda 2001 guidelines [16], by two experienced cytopathologists. An adjudicated final report was established and reported as 3 Negative for Intraepithelial Lesion or Malignancy (NILM), 7 Low-Grade Intraepithelial Lesions (L-SIL) and 9 High-Grade Intraepithelial Lesions (H-SIL).

### Claspin immunostaining

The immunohistochemistry (IHC) performed to detect claspin expression was carried out on 3 μm thick sections cut from formalin-fixed paraffin embedded blocks. We used a monoclonal antibody which was kindly provided by Professor T. Halazonetis (University of Geneva Switzerland), and directed against the amino-acidic residues 785–1056 of the full-length protein as previously described [7]. Antigen retrieval was carried out pretreating dewaxed and rehydrated slides in a water bath at 96 °C for 40 minutes in ethylenediamine tetracetic acid buffer (EDTA, pH 8.0). Immunoreactivity was revealed by means of a super sensitive streptavidin-biotin immunoperoxidase system (Novocastra, Menarini, Florence, Italy), using 3-amino-9-ethyl-carbazole as a chromogenic substrate. Nuclear staining, independent of intensity, was considered positive, excluding the basal layer in which proliferating cells are physiologically present. For each sample, we counted the positive nuclei in four to six selected High Power field (HPF, 400X magnification) representative of the lesion using an image analyzer (Eureka Interface, Menarini). A maximum of 200 immunoreactive nuclei/HPF were counted. For each sample, we calculated the mean number of the positive nuclei counted in all selected fields, thus obtaining a single value reported as nuclei/HPF value. For the purpose of the study, on the basis of the nuclei/HPF value, we distinguished 4 different categories of claspin reactivity: negative (0 or less than 1 immunoreactive nuclei/HPF), low-positive (1 to <20 immunoreactive nuclei/HPF), moderate-positive (20 to 80 immunoreactive nuclei/HPF), high-positive (more than 80 immunoreactive nuclei/HPF).

The immunocytochemical analysis of the 19 available cervico-vaginal samples was performed following the above described procedure, after fixing Thin-Prep slides in 10% buffered formalin for 20 minutes followed by water rinsing. Immunostaining was considered positive when at least one cell showed nuclear reactivity either among the superficial and intermediate typical cells or among the clearly atypical cells, independently of all other findings.

### HPV testing

The HR-HPV DNA detection was performed on cytological samples by the HR-HPV Hybrid Capture 2 (HC2) test (Qiagen, Milan, Italy), following the manufacturer’s recommendations. Before the HC2 test, 2 mL of each sample were processed using the HC2 Sample Conversion Kit (Qiagen). The HR-HPV HC2 assay detects the most common 13 HR-HPV types in cervical cancer: 16, 18, 31, 33, 35, 39, 45, 51, 52, 56, 58, 59 and 68. The HPV genotyping test was performed by the PCR based Linear Array HPV Genotyping kit (Roche Diagnostics, Italy), utilizing 250 μL of the residual liquid sample and following the manufacturer’s instructions. This assay is able to detect 37 high, intermediate and low risk HPV types (6, 11, 16, 18, 26, 31, 33, 35, 39, 40, 42, 45, 51, 52, 53, 54, 55, 56, 58, 59, 61, 62, 64, 66, 67, 68, 69, 70, 71, 72, 73, 81, 82, 83, 84, IS39 and CP6108). Only samples that were found positive to at least one of the 13 genotypes recognized by the HC2 test were considered to be HPV DNA positive by PCR. Moreover, because of the higher oncogenic potential displayed by HPV 16 and 18 types [13], we divided the positive results into two categories: 1) presence of HPV 16 and/or 18 sequences with or without other genotypes (16/18 positive), 2) detection of HR-HPV genotypes other than 16 and 18, as single or multiple infections (HR-HPV positive).

### Statistical analyses

Data were analyzed with SPSS statistical software version 17.0. (SPSS Inc., Chicago IL, USA). The associations between variables of interest were performed by the non-parametric Pearson Chi-Square test and the Kruskall-Wallis test, when appropriate. Analyses for trend were also carried out by using the Chi-square test for trend. A p-value < 0.05 was considered to be statistically significant. To evaluate the sensitivity and specificity for the presence of a CIN2+ lesion, the cases showing negative or low claspin expression were considered as negative whereas the cases with moderate or high claspin immunoreactivity were regarded to as positive.

## Results

### *In vitro* claspin expression

**Figure 1:**
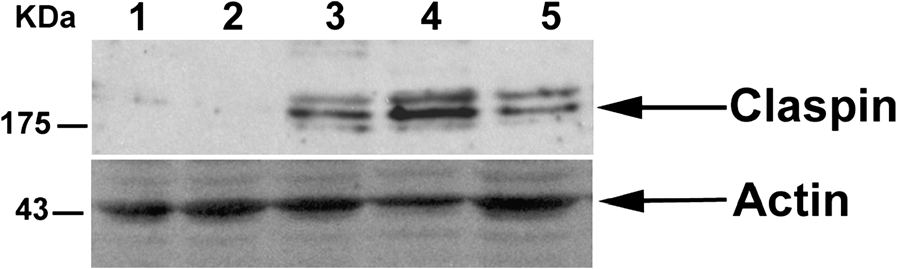
**Western blot analysis of cell lines.** Western blot analysis with a claspin specific antibody performed on NHEK (lane 1), HK-168 (lane 2), SiHa (lane 3), CaSki (lane 4), HeLa cell lines (lane 5). Actin was used as an internal control for sample loading. Because claspin activity is regulated by phosphorylation, the high molecular weight band might correspond to a phosphorylated form while the low molecular weight band might be due to protein degradation.

In order to investigate the correlation between claspin expression and uterine cervix transformation, we examined the expression of claspin by western blotting both in normal human keratinocytes (NHEK) and in a series of cell lines that resembled cervical malignancy at different stages of tumorigenesis (HK-168, SiHa, CaSki, HeLa). We found no claspin expression in the normal cell line and in the HK-168 cell line which has a basal/parabasal keratinocyte phenotype resembling the CIN2 stage [14], while its expression was evident in SiHa, CaSki and HeLa cervical carcinoma cell lines (Figure 1).

### Claspin expression in human cervical lesions

**Figure 2:**
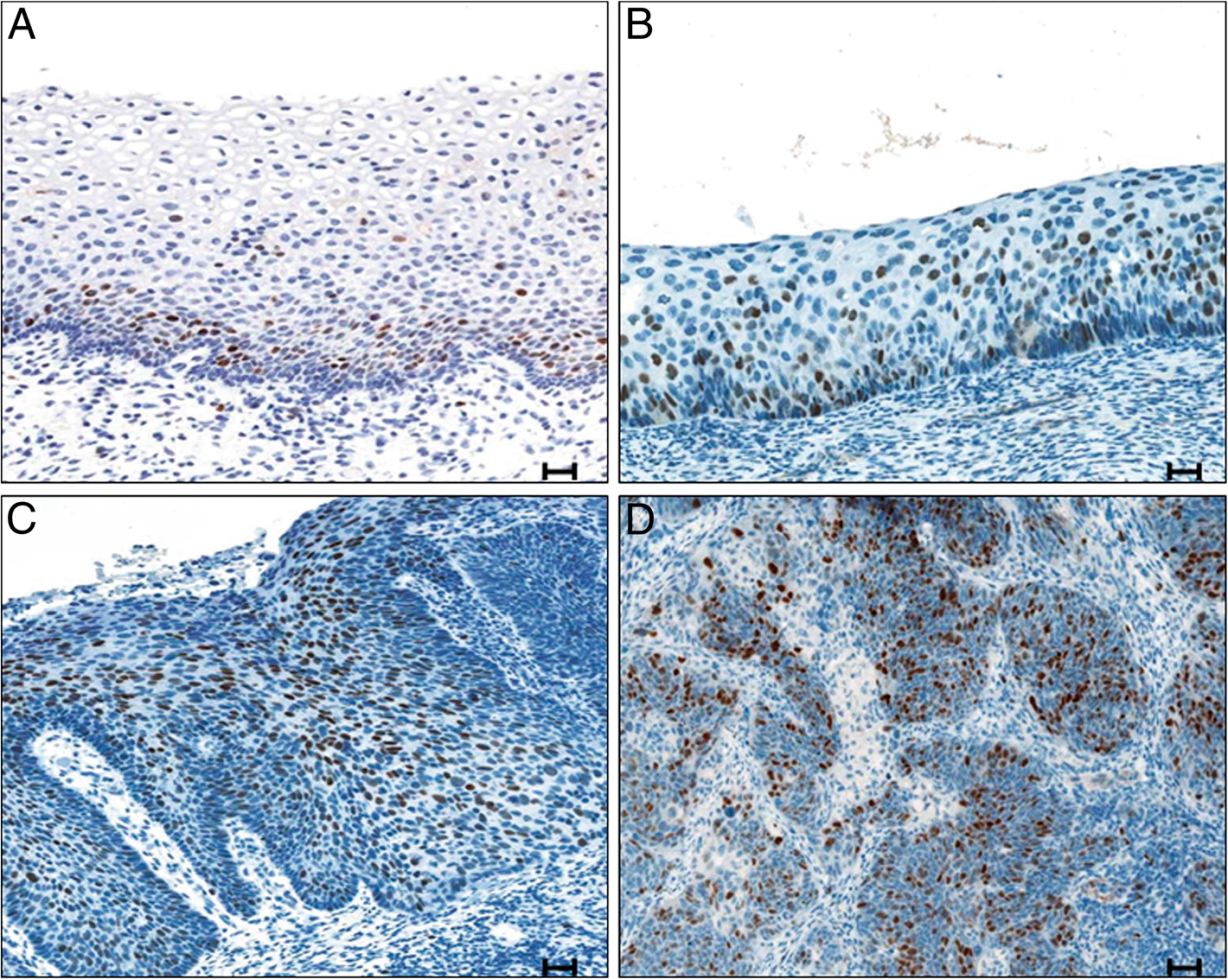
**Immunohistochemical staining of cervical biopsies for claspin expression.A**: CIN1 showing low immunoreactivity. **B**: CIN2 showing moderate immunoreactivity. **C**: CIN3 showing high immunoreactivity. **D**: Squamous Cell Carcinoma showing high immunoreactivity. Counterstaining with haematoxylin. Magnification 20X, scale bar = 30 μm. CIN Cervical Intraepithelial Neoplasia.

In order to investigate whether results obtained on the cell lines could be reproduced in human cervical tissue samples, and whether claspin expression could be linked to the severity of the lesions, we analyzed this biomarker by IHC (Figure 2) in a series of 9 WNL biopsies, 19 CIN1, 21 CIN2, 16 CIN3, and 15 SCC. We calculated the mean and the median value of the claspin-positive nuclei/HPF values within each diagnostic category (Table 1). We found that WNL tissues and CIN1 showed a very scanty mean positivity of less than 10 nuclei/HPF with a median value of 0.0. In contrast, we found a mean value of 46.8 ± 38.8 and a median of 36.5 in the CIN2 lesions, a mean value of 72.6 ± 46.7 and a median of 67.3 in the CIN3 lesions, and a mean value of 133.9 ± 75.8 and a median of 200.0 in the SCC. Therefore, the mean and median claspin-positive nuclei/HPF values consistently and significantly increased from CIN1 to SCC (p Kruskall-Wallis <0.0001). Of interest, the minimum claspin-positive nuclei/HPF value appeared to be independent of the severity of the histological diagnosis, whereas the maximum value showed a consistent increase from WNL to SCC samples.

**Table 1:** Distribution of claspin positive nuclei/HPF values by histological diagnosis

**Histology**	**Claspin-positive nuclei/HPF**	
	**Mean (±SD)**	**Median (Min-Max)**
WNL (n = 9)	0.4 (±0.8)	0.0 (0.0 -1.8)
CIN1 (n = 19)	7.4 (±13.8)	0.0 (0.0 -51.5)
CIN2 (n = 21)	46.8 (±38.8)	36.5 (1.0-148.6)
CIN3 (n = 16)	72.6 (±46.7)	67.3 (2.0-159.1)
SCC (n = 15)	133.9 (±75.8)	200.0 (11.8-200.0)

p(Kruskall-Wallis) < 0.0001.

HPF, High Power Field; SD, standard deviation; WNL, Within Normal Limit; CIN, Cervical Intraepithelial Neoplasia; SCC, Squamous Cell Carcinoma.

**Figure 3:**
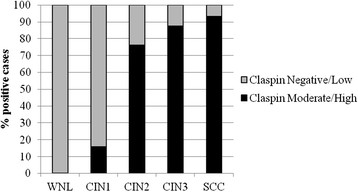
**Claspin immunoreactivity shown by histological diagnosis.** WNL Within Normal Limit.CIN Cervical Intraepithelial Neoplasia.SCC Squamous Cell Carcinoma.

Afterwards, according to the four-score categories of reactivity described in the Materials and Methods section, we compared the proportion of the negative/low positive and the moderate/high positive cases within each histological category (Figure 3). The 9 WNL tissues were mostly negative, only two cases showing low claspin positivity. Conversely, we observed a moderate/high positivity in 3 out of the 19 CIN1 lesions (15.8%), in 16 out of the 21 CIN2 lesions (76.2%), in 14 out of the 16 CIN3 lesions (87.5%), and in 14 out of the 15 SCC (93.3%). Therefore, starting from the WNL tissues through to carcinomas, we observed a constant and significant increase of claspin expression (pχ_trend_^2^ < 0.0001). In addition, claspin showed a high sensitivity for CIN2+ lesion (84.6%), and an even higher specificity (89.3%) (data not shown). However, due to the limited and nonrandomly selected series of cases, the accuracy indicator values should be interpreted with caution.

### Correlation between claspin immunoreactivity and HR-HPV infection

In order to investigate the correlation between claspin expression and HR-HPV infection, we compared the IHC findings with the HC2 results (Table 2). We observed a statistically significant correlation between moderate/high claspin expression and HR-HPV infection (p(*χ*^2^) <0.0001). In fact, 46 out of the 47 moderate/high claspin positive cases were HR-HPV positive (97.8%). In addition, 44 out of the 46 claspin positive/HR-HPV positive cases (95.6%) were CIN2+, whereas among the 23 claspin negative/HR-HPV positive cases we found only 8 CIN2+ lesions (34.7%). Therefore, among the HR-HPV positive cases, immunoreactivity for claspin seems to be associated with presence of a CIN2+ lesion.

**Table 2:** Correlation between claspin expression and HR-HPV infection assessed by HC2 assay, stratified by histological diagnosis

**Claspin expression n (%)**	**HR-HPV n (%)**	**WNL n = 9**	**CIN1 n = 19**	**CIN2 n = 21**	**CIN3 n = 16**	**SCC n = 15**
Negative/Low33 (41.2)	Negative 10 (30.3)	5	5	\	\	\
	Positive 23 (69.7)	4	11	5	2	1
Moderate/High47 (58.8)	Negative 1 (2.2)	\	1	\	\	\
	Positive 46 (97.8)	\	2	16	14	14

p(*χ*^2^) <0.0001.

HR-HPV, High Risk Human Papillomavirus; WNL, Within Normal Limit; CIN, Cervical Intraepithelial Neoplasia; SCC, Squamous Cell Carcinoma.

Furthermore, we performed a genotyping test in 58 out of the 69 HC2 positive cases. Overall, 3 cases were excluded from the analysis, because they showed positivity for one or more HPV types different from the 13 HR-HPV types officially detected by the HC2 test, probably due to the well documented cross-reactivity of this assay with other HPV types. All the remaining 55 cases were positive for one or more of the 13 HC2 HR-HPV types. In particular, as shown in Table 3, 30 out of 55 specimens presented the HPV16 and/or 18 types (54.5%), while 25 displayed other HR genotypes (45.5%). Comparing the distribution of claspin immunostaining with the PCR findings, we found no significant association between claspin immunoreactivity and HPV16/18 infection. In fact, even though 22 out of the 30 HPV 16/18 positive cases showed moderate/high claspin immunostaining (73%), we found a not statistically significant difference in moderate/high immunoreactivity rate in comparison with the samples infected by other HR-HPV genotypes (16 out of 25 cases, 64%; p = 0.65).

**Table 3:** Association between claspin expression and HPV genotyping in 55 HC2-positive samples

**HPV genotyping**	**Claspin expression**		
	**Negative/Low**	**Moderate/High**	**Total**
HPV 16/18 positive^*^	8 (27%)	22 (73%)	**30 (100%)**
HR-HPV positive^†^	9 (36%)	16 (64%)	**25 (100%)**
Total	17 (31%)	38 (69%)	55 (100%)

^*^ Presence of HPV16 and/or 18 with or without other HPV types.

^†^ Presence of one or more HR-HPV types other than HPV16 and 18, among the HR types included in the HC2 test.

p(*χ*^2^) = 0.65.

### Claspin expression in cervical cytology

In order to verify whether claspin detection was feasible on cervical cytology, we evaluated its expression in the 19 available cervico-vaginal samples out of the 80 corresponding histological specimens tested, as specified in the Materials and Methods section. As shown in Table 4, despite the small number of specimens, claspin expression seems to correlate with the severity of the cellular abnormalities. In fact, only one NILM sample was claspin positive, while 4 out of the 7 L-SIL (57%) and all the 9 H-SIL (100%) showed claspin positive nuclei. Moreover, we found that all the 14 claspin immunoreactive cases were also HC2 positive and 13 out of these had a histologically confirmed lesion (4 L-SIL: 2 CIN1 and 2 CIN2; 9 H-SIL: 6 CIN2 and 3 CIN3; data not shown). It is worth noting that the only claspin positive NILM case was HC2 positive. However, due to the limited number of cytological cases analyzed we could not perform any statistical analysis to determine whether the observed differences were statistically significant.

**Table 4:** Distribution of claspin expression by HR-HPV infection and cytological categories in 19 cervico-vaginal samples

**Claspin expressionn (%)**	**HR-HPV n (%)**	**NILM n = 3**	**L-SIL n = 7**	**H-SIL n = 9**
Negative 5 (26.3)	Negative 3 (60.0)	2	1	\
	Positive 2 (40.0)	\	2	\
Positive14 (73.7)	Negative 0 (0.0)	\	\	\
	Positive 14 (100)	1	4	9

HR-HPV, High Risk Human Papillomavirus; NILM, Negative for Intraepithelial Lesion or Malignancy; L-SIL, Low Grade Squamous Intraepithelial Lesion; H-SIL, High Grade Squamous Intraepithelial Lesion.

## Discussion

In this study, we analyzed claspin expression in a series of tissue samples which covered the different phases of uterine cervix transformation. This multistep process represents an important model of tumor development and progression. In fact, differently from the majority of human solid tumors, its causative agent, namely the HR-HPV, is well defined. Nevertheless, although the steps preceding cervical cancer have been extensively studied and are known to be linked to the active transcription of the E6 and E7 viral oncogenes, only a few biomarkers of active infection have been identified to date. Claspin is a critical regulator of the ATR/Chk1 signalling axis in the G2 DNA damage checkpoint [8] and it might represent a novel marker of uterine cervix transformation. In fact, it has been demonstrated that the viral oncoproteins activate the proliferation pathway even in presence of DNA damage, concomitantly affecting a number of cellular proteins, including claspin. Therefore, claspin up-regulation may represent a suitable marker of altered cellular proliferation due to HR-HPV infection. In cervical pathology, other putative biomarkers linked to proliferation control, have been investigated, e.g., Ki67, PCNA, minichromosome maintenance protein (MCM), and cyclins A and E [12,13,17]. These proteins, which are normally active during S-phase entry, are up-regulated during HPV infection. Nevertheless, none of these biomarkers has been validated in a clinical setting so far. The cyclin-dependent kinase inhibitor p16^INK4a^, involved in cell cycle control, has also been found overexpressed in pre-neoplastic and neoplastic lesions of the uterine cervix as a consequence of the infection by HR-HPV [11]. Although p16 is one of the most promising biomarker, and is being investigated in clinical settings [18], the immunohistochemical evaluation of p16 usually presents a limited reproducibility due to the lack of standardized criteria for the interpretation of immunostaining [19].

To our knowledge, this is the first study in which claspin expression has been analyzed in histological as well as cytological cervical specimens with the aim to establish whether this marker is related to the severity of cervical lesions and to HR-HPV infection. This approach represents the first step of the framework proposed by Arbyn to assess the potential use of a biomarker as a screening tool for cervical cancer [20].

When we firstly evaluated claspin level in a series of different cell lines, we evidenced an up-regulation of the protein only in the cervical cancer cell lines SiHa, CaSki and HeLa. Afterwards, in human cervical tissues, we observed a significant positive correlation between the number of claspin immunoreactive nuclei/HPF and the severity of the lesion. In particular, while the minimum number of claspin-positive nuclei/HPF was almost the same in all the histological categories, the maximum value significantly increased from normal cervix to carcinomas. This latter value could be particularly useful in clinical practice contributing to define the risk of progression of each lesion. In fact, despite a marked overlap between the different entities, claspin high expression may identify those CIN1 prone to progression. On the other hand, high grade lesions displaying low expression may be prone to regression. However, only a prospective study may clarify the prognostic role of claspin. In a follow up study, a parallel analysis of p16 and claspin expression may be of interest, since p16 has been proposed as a prognostic biomarker. Our findings are mostly in agreement with recent data [7] showing that claspin levels are higher in human colon, lung, bladder and breast carcinomas than in the corresponding autologous normal tissues, even though we did not analyze autologous normal tissues. In the present study, we took our analysis one step further investigating whether claspin expression was differently modulated in the dysplastic lesions that precede the cancer. This methodological approach, together with the claspin reactivity score applied in this study, may provide more useful biological information to be translated into clinical practice.

Concerning the correlation between claspin and HR-HPV infection, in our series of samples we found a statistically significant association between the protein expression and HR-HPV infection detected by the HC2 test, independently of the HR-HPV genotypes. This association might have a particular clinical value, since the 13 HR-HPV types detected by the HC2 test are responsible for the majority of the high grade lesions as well as cervical carcinomas (97% and 93.6% respectively) [21,22]. Therefore, we can hypothesize that claspin identifies almost all the high grade lesions and cancers. Interestingly, our study seems to evidence a correlation between claspin positivity and the presence of a CIN2+ lesion among the HR-HPV positive cases. There are no studies regarding the interaction between claspin expression and HPV infection *in vivo*, and only one *in vitro* study has been published so far [8]. The authors provide evidence that both HPV-positive and negative cervical cancer cell lines show increased baseline levels of claspin. Yet, HPV16-positive cell lines show an accelerated proteolitic turnover which is probably due to HPV E7 oncoprotein. Therefore, the authors suggest that HPV16-infected cells can also enter mitosis in the presence of unrepaired DNA, probably overcoming the DNA damage checkpoint control. In this case, the accumulation of claspin attempts to restore cell cycle control reinforcing the G2-M checkpoint. These findings appear to be consistent with our results, and open the need to delve deeper into the matter by carrying out a more accurate investigation of the correlation between claspin and HR-HPV infection.

Focusing on cervical cytology, we found that claspin positivity was associated with HR-HPV positivity and consistently increased with the severity of cellular abnormalities. Immunoreactivity was mainly found among cases with a histologically confirmed diagnosis. These findings, although preliminary, may have relevant clinical implications, mainly in the management of HPV positive cases. In fact, the immunocytochemical detection of this biomarker may represent a novel and useful tool which is capable of identifying those HR-HPV infections associated to a lesion. In fact, HR-HPV testing, despite its high sensitivity, is limited by a low specificity in detecting high grade lesions, since the majority of HPV infections are transient and not linked to histologically confirmed cervical dysplasia [23].

## Conclusions

Our findings indicate that *in vivo* claspin expression is significantly related to HR-HPV infection and lesion grade both in histological and cytological samples. Therefore, the analysis of claspin expression could be clinically relevant in the diagnosis of HPV-related cervical lesions, in particular when applied to cervico-vaginal cytology. Moreover, giving information on the proliferation rate of each lesion, claspin immunostaining may contribute to the evaluation of progression risk, thus being helpful in patient management. Nevertheless, only large prospective studies may clarify the true clinical usefulness of claspin expression in distinguishing lesions with different progression potential.

## Abbreviations

HR-HPV, High risk human papillomavirus; WNL, Within normal limits; CIN, Cervical intraepithelial neoplasia; SCC, invasive squamous cell carcinomas; NILM, Negative for intraepithelial lesion or malignancy; L-SIL, Low-grade intraepithelial lesions; H-SIL, High-grade intraepithelial lesions; IHC, Immunohistochemistry; HPF, High power field; HC2, Hybrid capture 2.

## Competing interests

The Authors declare that they have no competing interests.

## Authors’ contributions

MB conceived of the study, wrote the manuscript and performed literature search and review; AM performed Western blotting analyses and revised the manuscript; AV and MGD evaluated the cytological and molecular findings and addressed relationships between molecular and pathological findings; FR performed immunohistochemical analysis and HPV testing; IT performed the statistical analyses; MC and EP performed histological analyses; GV helped in the enrollment of cases; MM participated in study design, supervised all the activities, and helped write the manuscript. All authors read and approved the final manuscript.
